# Legislation

**Published:** 1902-05

**Authors:** 


### LEGISLATION.

### Official Copy Reprint.

Senate, No. 76, State of New Jersey, introduced February 17, 1902, by Mr. McKee. Referred to Committee on Miscellaneous Business.

An Act to regulate the practice of veterinary medicine, surgery, and dentistry in the State of New Jersey, to license veterinarians, and to punish persons violating the provisions thereof.

*Be it enacted by the Senate and General Assembly of the State of New Jersey:*

1. The Governor shall appoint a Board of Examiners, to be known as the State Board of Veterinary Medical Examiners, said Board to consist of five members, who shall be persons of recognized professional ability and honor in the veterinary profession in this State, and who shall have practised veterinary medicine and surgery for at least five years immediately preceding such appointment. The term of office of the members of said Board shall be three years, or until their successors are appointed and duly qualified: *Provided, however,* That the members of the Board first appointed shall serve as follows: one for one year, two for two years, and two for the full term of three years, commencing on the first Monday of May, one thousand nine hundred and two, and thereafter each member shall be appointed for the term of three years. Each appointee shall, before assuming the duties of the office, and within thirty days after the receipt of his commission, take, subscribe, and file, in the office of the Secretary of State, the oath or affirmation of office. The Governor shall fill vacancies from death or otherwise for unexpired terms, and may remove any member of said Board for continued neglect of the duties required by this Act, for incompetence, unprofessional or dishonorable conduct.

2. The first meeting of the Examining Board shall be held on the first Monday in May, one thousand nine hundred and two, suitable notice in the usual form being given, with the notice of their appointment, by the Secretary of State, to each of the members thereof, specifying the time and place of said first meeting. At the first meeting of the Board an organization shall be effected by the election from their own membership of a president, a secretary, and a treasurer. It shall have a common seal, and its president shall be empowered to issue subpœnas and to administer oaths in taking testimony in any matter pertaining to the duties of said Board; it shall make and adopt all necessary rules, regulations, and by-laws not inconsistent with the laws of this State or of the United States whereby to perform the duties and to transact the business required under the provisions of this Act.

3. Said Board shall hold two or more meetings for examinations at the capitol building of this State each year, due notice of which shall be made public at such time as they shall determine. At all meetings a majority of the members of the Board shall constitute a quorum, but the examination of applicants for license may be conducted by a committee of one or more members duly authorized by said Board. Said Board shall examine all diplomas as to their genuineness, and each applicant for a license shall submit to a theoretical and practical examination, said examination to be written, oral, or both. Such examination shall include the following subjects: veterinary anatomy, physiology, chemistry, surgery, dentistry, practice of veterinary medicine, obstetrics, pathology, bacteriology, diagnosis, materia medica, therapeutics, pharmacy, zoötechnics, sanitary medicine, hygiene, meat- and milk-inspection, and veterinary jurisprudence.

4. Said Board shall issue forthwith to each applicant who has passed such examination successfully, and who shall have been adjudged duly qualified for the practice of veterinary, medicine, surgery, and dentistry, a license to practise the same in the State of New Jersey. Such license issued pursuant to this Act shall be subscribed by the president and secretary of the Board of Veterinary Medical Examiners. It also shall have affixed to it, by the person authorized to affix the same, its corporate seal. Before said license shall be issued it shall be recorded in a book kept in the office which said Board shall establish for the purpose of carrying out the provisions of this Act, and the number of the book and the page therein containing said recorded copy shall be noted on the face of said license. Such records shall be open to public inspection, with proper restrictions as to their preservation.

5. Upon presenting to the Board a certified copy of a court record showing that a practitioner of veterinary medicine, surgery, or dentistry has been convicted of a felony or misdemeanor, that fact may be noted upon the record of licenses, and the license and registration shall be marked cancelled. Any person whose license shall be so cancelled shall be deemed as an unlicensed person, and, as such, subject to the penalties prescribed for other unlicensed persons who practice veterinary medicine, surgery, or dentistry in this State.

6. From and after the first Monday in May, one thousand nine hundred and two, any person not hereinbefore registered to practise veterinary medicine, surgery, and dentistry in this State, or desiring to enter upon such practice, shall deliver to the secretary of the Veterinary Medical Board, upon a payment of a fee of ten dollars, a written application for license, together with satisfactory proof that the applicant is more than twenty-one years of age, is of good moral character, has obtained a competent school education, and has received a diploma conferring the degree of veterinary medicine from some legally incorporated veterinary college or university of the United States, or a diploma or license conferring the full right to practise all the branches of veterinary science in some foreign country (which, in the opinion of said Board, was in good standing at the time of issuing said diploma). Applicants who shall have received their degree in veterinary medicine after the first Monday of May, one thousand nine hundred and two, must have pursued the study of veterinary medicine for at least three years, including three regular courses of lectures of at least six months each in different years, in some legally incorporated veterinary college or university, prior to the granting of said diploma or foreign license. Such proof shall be made, if required, upon affidavits. Upon making the said payment and exhibiting the before-named proof the Examining Board, if satisfied with the same, shall issue to such applicant an order for examination. In case of failure at such examination the candidate, after the expiration of six months and within two years, shall have the privilege of a second examination by the Board of Veterinary Medical Examiners, without the payment of an additional fee: *And it is further provided*, That applicants examined and licensed by the State Board of Veterinary Medical Examiners of other States, on payment of a fee of ten dollars to the Examining Board of this State, and on filing in the office of said Board a copy of said license, certified by the affidavit of the president or secretary of the Board of such other State, showing also that the standard of the examination and other requirements adopted by that State Board of Veterinary Medical Examiners is substantially the same as that provided for by this Act, shall, without further examination, receive a license conferring upon the holder thereof all the rights and privileges provided for by sections 4 and 6 of this Act.

7. From and after the first Monday in May, one thousand nine hundred and two, no person shall enter upon or continue the practice of veterinary medicine, surgery, or dentistry, in any of their branches, in the State of New Jersey, unless he has complied with the provisions of this Act, and shall have exhibited to the clerk of the county in which he desires to practise veterinary medicine, surgery, or dentistry a license duly granted to him as hereinbefore provided; whereupon he shall be entitled, upon the payment of one dollar, to be duly registered in the office of the clerk of the court of common pleas in said county. Any person using any title or degree appertaining to the veterinary profession, or practising veterinary medicine, surgery, or dentistry, in any of their branches in this State after the first Monday in May, one thousand nine hundred and two, without being licensed and registered in conformity with the provisions of this Act, or otherwise violating any of its provisions, shall be guilty of misdemeanor, and upon conviction thereof shall be punished for the offense by a fine of not less than one hundred dollars, or by imprisonment in the county jail for a period of not less than thirty days, or by both fine and imprisonment, and for each subsequent offense the punishment shall be double that of the preceding one; and it shall be the duty of the respective district attorneys of the counties of this State to prosecute violations of the provisions of this Act.

8. It shall also be lawful for the said Board to institute civil proceedings in any court of competent jurisdiction against any person, company, or association for the violation of any of the provisions of this Act. Such proceedings shall be brought in an action on contract, and upon conviction thereunder the person, company, or association so convicted shall be liable to a fine, which shall be the same amount fixed in the preceding section of this Act; and all fines and penalties collected by any court under the provisions of this section of this Act shall be paid over to the treasurer of this Board, to be received and disbursed by him in accordance with the provisions of this Act.

9. Nothing in this Act shall be construed to interfere with or punish veterinarians in the United States army, or in the United States Bureau of Animal Industry, while so commissioned, or any lawfully qualified veterinarians residing in other States or countries meeting registered veterinarians of this State in consultation, or any veterinarian residing on the border of a neighboring State and duly authorized under the laws thereof to practise veterinary medicine or surgery therein whose practice extends into the limits of this State: *Provided,* That such practitioner shall not open any office or appoint a place to meet patients or receive calls within the limits of New Jersey; and nothing in this Act shall be construed to prohibit the practice of veterinary medicine, surgery, or dentistry, by any practitioner who shall have been registered in any county in this State before the first Monday in May, one thousand nine hundred and two, and one such registry shall be sufficient warrant to practise veterinary medicine, surgery, or dentistry in any county in this State. Nothing in this Act shall apply to persons gratuitously treating animals in cases of emergency: *Provided,* They do not represent themselves to be veterinarians or use any title or degree appertaining to the practice thereof.

10. The expenses of said Board and of the examinations shall be paid from the license fees and fines above provided for, and if any surplus remains the same may be distributed among the members of said Board as a compensation for their services as members, but otherwise they shall receive no compensation whatever.

11. All Acts or parts of acts, general or special, now existing not in accordance with the provisions of this Act or inconsistent herewith be and the same are hereby repealed.

12. This Act shall take effect immediately.

Approved March 17, 1902.

---

### PENNSYLVANIA STATE BOARD OF VETERINARY MEDICAL EXAMINERS.

THE annual meeting of this Board for the examination of applicants desiring to obtain a license to practise veterinary medicine and surgery in the State of Pennsylvania will be held in Philadelphia, Pa., June 16 and 17, 1902.

W. HORACE HOSKINS,
Secretary.

3452 LUDLOW ST., PHILADELPHIA, PA.

### NEW STATE BOARD OF EXAMINERS.

THE Governor of New Jersey has appointed the following members of the profession on the Board: Drs. Wm. Herbert Lowe, Paterson, N. J.; Whitfield Gray, Newton; T. E. Smith, Jersey City; T. Earle Budd, Orange, and T. B. Rogers, Woodbury, N. J. President Lowe announces that an examination for license to practise veterinary medicine, surgery, and dentistry will be held in the State House, Trenton, on Tuesday, June 24, 1902.

